# Association of Strawberries and Anthocyanidin Intake with Alzheimer’s Dementia Risk

**DOI:** 10.3390/nu11123060

**Published:** 2019-12-14

**Authors:** Puja Agarwal, Thomas M Holland, Yamin Wang, David A Bennett, Martha Clare Morris

**Affiliations:** 1 Rush Institute of Healthy Aging, Department of Internal Medicine, Rush University Medical Center, Chicago, IL 60612, USA; thomas_holland@rush.edu (T.M.H.); yamin_wang@rush.edu (Y.W.); martha_c_morris@rush.edu (M.C.M.); 2 Rush Alzheimer’s Disease Center, Rush University Medical Center, Chicago, IL 60612, USA; david_a_bennett@rush.edu; 3 Department of Neurological Sciences, Rush University Medical Center, Chicago, IL 60612, USA

**Keywords:** diet, flavonoids, pelargonidin, proanthocyanidins, cyanidins, APOE-ɛ4

## Abstract

Background: Strawberries have been identified to have antioxidant and anti-inflammatory properties that improve neuronal function and cognition, mostly in animal studies. It is unknown if the consumption of strawberries or related bioactives may reduce the risk of Alzheimer’s dementia risk. Material and Methods: The study was conducted in 925 participants, aged 58–98 years of the Rush Memory and Aging Project. Participants were dementia-free at baseline, completed a food frequency questionnaire, and had at least two annual neurological evaluations. The diagnosis of Alzheimer’s dementia was based on structured clinical neurological examination and standardized diagnostic criteria. The association of strawberry intake and incident Alzheimer’s dementia was analyzed using proportional hazard models adjusted for age, sex, education, physical activity, participation in cognitive activities, APOE-ɛ4 genotype, dietary intake of other fruits, and total calorie intake. Results: A total of 245 participants developed Alzheimer’s dementia over the mean follow-up of 6.7 (±3.6) years. Higher strawberry intake was associated with reduced risk of Alzheimer’s dementia (HR = 0.76, 95% CI: 0.60–0.96). In separate adjusted models, highest vs. lowest quartile intakes of Vitamin C (HR = 0.64, 95% CI: 0.45, 0.92), Pelargonidin (0.63, 95% CI: 0.43, 0.92), total anthocyanidins (0.69, 95% CI: 0.48, 0.99), and total flavonoids (0.67, 95% CI: 0.46, 0.98) were each associated with lower Alzheimer’s dementia risk. These associations remained after further adjustment for cardiovascular conditions. Conclusion: Consumption of strawberries and foods rich in vitamin C, pelargonidin, anthocyanidins, and total flavonoids may reduce the risk of Alzheimer’s dementia.

## 1. Introduction:

Alzheimer’s dementia in older adults is associated with loss of well-being, other disabilities, and motor decline [1,2,3]. The potential mechanistic link for these conditions is age-related alterations in the brain due to increased oxidative stress and inflammation [4,5,6]. Currently, with limited treatment options, modifiable risk factors, such as diet, are important to explore as potential prevention strategies. Previous studies have shown diets rich in antioxidant nutrients (e.g., vitamin E, lutein, and beta-carotene) and flavonoids (i.e., total intake of different subclasses-anthocyanidins, flavan-3-ols, flavonols, flavones, and proanthocyanidins) reduce the risk of Alzheimer’s dementia [7,8,9,10]. A limited number of in-vitro, animal, and controlled human feeding studies suggest that strawberries have anti-inflammatory and anti-oxidative properties, possibly attributed to their high content of flavonoids, anthocyanidins, and vitamin C [11]. Strawberries have been identified to improve neuronal function, cognition, and some motor outcomes, mostly in animal studies [12,13]. Rats who were fed chow infused with strawberry extract had better neuronal signal transduction, higher cognitive and motor performance, and increased brain neurons compared to controls [12,14,15,16]. To our knowledge, only the Nurses’ Health Study investigated this in humans and reported slower cognitive decline among older women who consumed strawberries more than two times per week when compared to those consuming less than once a week [17]. In the present analysis, we investigated the association between strawberry consumption and the risk of Alzheimer’s dementia in a community cohort of older adults. To further understand the possible mechanistic link, we also assessed the relation to dementia of those dietary bioactive for which strawberries are a rich food source.

## 2. Material and Methods

### 2.1. Study Population

The analytical sample is drawn from an ongoing longitudinal cohort study, the Rush Memory, and Aging Project [18]. From 1997 to 2018 the study enrolled 2152 residents of retirement communities and senior public housing. All agreed to annual clinical evaluations and brain donation at the time of death. In 2004 when the dietary assessments were introduced to the study, 127 of Memory and Aging Project (MAP) participants were deceased. Since then, 1628 participants have completed at least one dietary assessment. Out of which, 567 food frequency questionnaires (FFQs) are under processing for the complete nutrient analysis. For the present analyses, we only considered participants with the complete diet data (including total calories and various nutrient intake, *n* = 1061). We further excluded those with dementia at baseline (*n* = 36), less than two cognitive assessments (*n* = 92), or missing data on model covariates (*n* = 8). Thus, our analytical sample consisted of 925 participants with a follow-up of 6.7 (±3.6) years. The baseline characteristics of the analytical sample were similar to the overall MAP participants enrolled in the cohort (Supplementary Table S1). All subjects gave their informed consent for inclusion before they participated in the study. The study was conducted in accordance with the Declaration of Helsinki, and the protocol was approved by the Ethics Committee of Rush University Medical Center (IRB number: 9020402).

### 2.2. Alzheimer’s Dementia

At each annual assessment, participants undergo a three-stage process for Alzheimer’s dementia diagnosis including computerized scoring, clinical judgment by a neuropsychologist, followed by a diagnostic classification by a clinician [19]. The neurological exams include uniform, structured, clinical evaluation for the battery of 21 cognitive tests. Of these, 11 are scored by education-adjusted impairment rating and summarized as impairment in five cognitive domains. A clinical judgment is then done by a neuropsychologist (blinded to participants’ demographics other than education) based on the impairment rating and other clinical information. The final diagnostic classification of Alzheimer’s dementia is given by an experienced clinician after reviewing all the available data, based on criteria of the joint working group of Neurological and Communicative Disorders and Stroke and the Alzheimer’s disease and Related Disorders Association (NINCDS-ADRDA) [20].

### 2.3. Diet Assessment

Diet was assessed annually using a 144-item food frequency questionnaire (FFQ) that had been previously validated in older Chicago residents [21,22]. Strawberry consumption was based on the first obtained FFQ and the following response options: never or less than once per month, 1–3 times per month, once per week, and 2–4 times per week. The correlation between strawberry intakes reported at baseline and the last visit before death were moderately strong (r = 0.50, *p* =< 0.0001), and there was not much change in reported mean intake (0.64 servings/day at baseline and 0.70 servings/day at the last follow-up). We also assessed the variation in the reporting of strawberry intake by season and found no significant differences in intake by the month of FFQ administration. Total calories and nutrient intakes were based on United States Department of Agricultural (USDA) National Nutrient Database [23]. Food levels of flavonoids and flavonoid subclasses were based on the Nutrition Coordinating Center Flavonoid and Proanthocyanidin Provisional Table from the University of Minnesota [24], which draws heavily on the USDA Database for the Flavonoid Content of Selected Foods, Release 3.3 (March 2018,) and The USDA Database for the Proanthocyanidin Content of Selected Foods, Release 2.1 (March 2018), with additional data from study publications. Calorie, nutrient, and flavonoid intake per day were estimated from responses to all 144 FFQ items. For each food, the nutrient/flavonoid levels in one portion size were multiplied by the frequency of intake reported, and the values for all foods were summed together. The portion sizes were described as either natural portion size (e.g., one banana) or mean portion sizes reported by the oldest men and women in national survey data collected by 24 h dietary recalls. The bioactive of interest in the present analysis were those primarily present in strawberries, including vitamin C (58.8 mg/100 gm strawberries), anthocyanidins (mainly Pelargonidin (24.9 mg/100 gm strawberries), cyanidin (1.7 mg/100 gm strawberries), total proanthocyanidins (105.2 mg/100 gm strawberries), and total flavonoids (138.7 mg/100 gm strawberries). All the bioactive were assessed in food sources only, whereas vitamin C was assessed as vitamin C from food sources and total vitamin C (food sources + supplements). 

### 2.4. Covariates

Based on previous literature various dietary and non-dietary factors were selected as covariates to assess the association between strawberry/bioactive association with Alzheimer’s dementia risk. Dietary intake levels of total energy (kcal/day), other fruits intake (servings /day), leafy green vegetables (servings /day), and seafood (servings/day) were based on the baseline FFQ responses. Other fruits included all fruits other than strawberries in the questionnaire (including grapes, banana, melon, apple, pear, orange, peach, plum, and apricots). Leafy green vegetable consumption included three food items (spinach, kale/collard/greens, and lettuce) and seafood intake included tuna sandwich, fish sandwich, fish as a main dish, and shrimp/lobster/crab. Non-dietary factors, including age (in years), were computed based on the self-reported date of birth and date of baseline FFQ and education was self-reported years of education. APOE-ɛ4 genotyping was performed using the sequencing as described earlier [25]. Participating in cognitive activities was computed as the average frequency rating, based on a 5-point scale, of seven activities (playing games, writing letters, visiting the library, reading newspapers, magazines, and books) [26]. Physical activity (hours/week) was computed based on self-reported minutes spent over the past two weeks on five activities, including walking for exercise, yard work, calisthenics, biking, and water exercise [27]. Cardiovascular conditions included hypertension, diabetes, myocardial infarction, and stroke. Hypertension was determined by self-reported medical diagnosis, current use of antihypertensive medication, or a measure of systolic/diastolic blood pressure of ≥160/90 mm Hg [28]. Myocardial infarction was self-reported or use of cardiac glycosides (e.g., digoxin, Lanoxin, etc.) as medication. Diabetes was also either a self-reported medical diagnosis or indicated by the current use of diabetic medication [29]. History of stroke was based on self-reported questions, cognitive testing, interviews with participants, and neurological examination (when available) [30]. At the time of annual assessment, all medications taken within the previous two weeks are inspected and recorded by the interviewer. 

### 2.5. Statistical Methods

Baseline characteristics of the study population were defined using mean (±SD) for continuous variables and percentages for dichotomous variables. Correlations between strawberry intake and various bioactive of interest were assessed using Spearman’s correlation coefficient. To determine the association between strawberry consumption and related nutrients and bioactive with incident Alzheimer’s dementia, we used cox-proportional hazard models programmed in SAS version 9.4 (SAS Institute, Cary, NC) that generated hazards ratio as the estimates. To assess the effect of one serving change in strawberry intake, the frequency of strawberry consumption was modeled as a continuous variable with values of 0 (never or less than once a month); 0.5 (1–3 times/month); 1.0 (once per week), and 2 (2–4 times/ week). Nutrients and bioactives were energy-adjusted using the residual regression method [31] and modeled in quartiles with the lowest quartile as the referent category. The basic models were adjusted for age, sex, education, physical activity, participation in cognitive activities, APOE-ɛ4 allele (any versus none), dietary intake of other fruits, and total calorie intake. Models were further adjusted for a) other dietary factors that are known to be associated with Alzheimer’s dementia (leafy greens and seafood intake), b) total vitamin E intake (known to reduce Alzheimer’s dementia risk). Further to assess if the strawberry or bioactive association with Alzheimer’s dementia is mediated by its effect on cardiovascular conditions, we adjusted the models for hypertension, diabetes, myocardial infarction, and stroke. Additionally, a test of the linear trend was assessed for each by assigning the median quartile intake level to all those in a given quartile and modeling as a single categorical variable. Tests for potential effect modification by age, sex, education, APOE-ɛ4 allele were conducted by including a multiplicative term between the dietary exposure variable and the effect modifier of interest (*p* ≤ 0.05). 

## 3. Results

The analytical sample of 925 participants was on average 81 (±7.2) years of age, 75% female, 98% White, had a mean educational level of 15 (±3) years, and 21.5% had an APOE-e4 allele. A total of 245 participants developed Alzheimer’s dementia over the mean follow-up of 6.7 (±3.6) years. Strawberry intake ranged from 0 to 2 servings/week (mean intake of the population: 0.64 servings/week). The baseline characteristics of participants consuming strawberries more than once a week were similar to those consuming strawberries rarely or a few times/month (Table 1). Strawberry consumption was weakly correlated with the consumption of other healthy foods, including other fruits, leafy green vegetables, seafood, beans/legumes, olive oil, and nuts (ρ = 0.11 to 0.26, *p* < 0.0001). Strawberry consumption was highly correlated with dietary intake of pelargonidin (ρ = 0.94), one of the primary anthocyanidins found in strawberries; and in this population, strawberries contributed 75% of the total dietary pelargonidin intake. Intake of proanthocyanidins (ρ = 0.39), cyanidins (ρ = 0.42), total flavonoids (ρ = 0.26), and vitamin C (ρ = 0.25) were moderately associated with strawberry intake (Supplementary Table S2).

### 3.1. Strawberries and Alzheimer’s Dementia Risk

**Figure 1:**
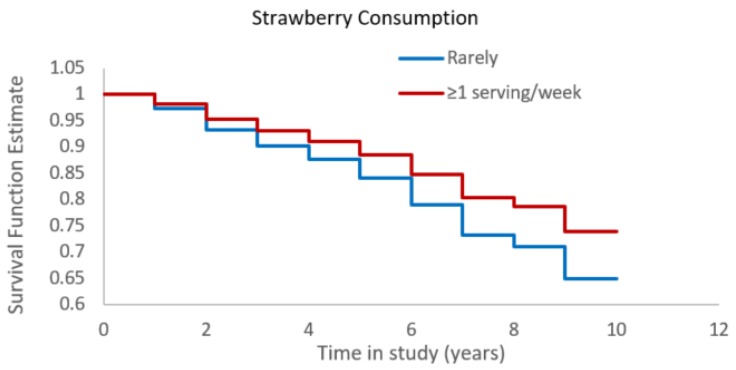
Strawberry consumption and Alzheimer’s dementia risk (Cox-proportional hazards model adjusted for age, sex, education, physical activity, participation in cognitively stimulating activities, APOE-ɛ4, other fruits intake, and total calories).

We found that one serving more in strawberry intake was associated with a 24% reduced risk of Alzheimer’s dementia (Table 1), when controlled for age, sex, education, physical activity, participation in cognitive activities, Apo-ɛ4 status, dietary intake of other fruits, and total calorie intake. Additionally, we controlled for other foods that reported to be associated with better cognition, including leafy green vegetables and seafood. The association of strawberry intake and Alzheimer’s dementia risk was not materially different from these further adjustments (HR = 0.79, 95% CI: 0.62, 0.99). To investigate whether the association was mediated by dietary effects on cardiovascular conditions, we further adjusted for presence of hypertension, diabetes, myocardial infarction, and stroke but there was little change in the effect estimates (HR = 0.75, 95% CI: 0.60, 0.95), indicating that the association is not mediated by cardiovascular conditions. We also assessed any confounding by total vitamin E intake and found no difference in the association (Table 2). Overall, participants consuming one or more servings of strawberries per week had a 34% lower risk of developing Alzheimer’s dementia when compared to those consuming none or less than once per month (HR = 0.66 (95% CI: 0.46, 0.95; Figure 1).

### 3.2. Bioactives and Alzheimer’s Dementia Risk

**Figure 2:**
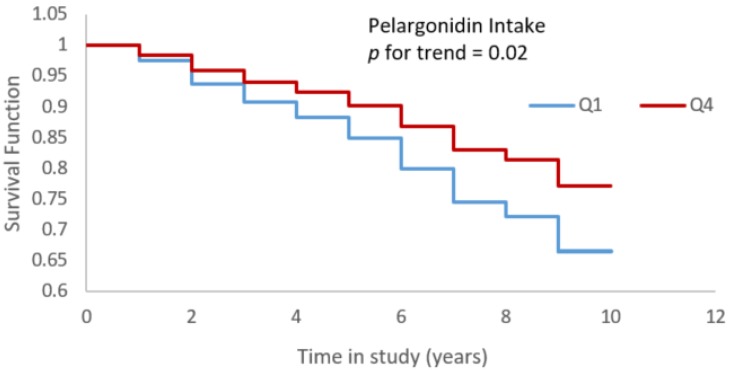
Higher pelargonidin intake associated with reduced Alzheimer’s dementia risk (Cox-proportional hazards model adjusted for age, sex, education, physical activity, participation in cognitively stimulating activities, and APOE-ɛ4; Quartile 4 vs. Quartile 1).

Strawberries are rich sources of vitamin C and other bioactives, including anthocyanidins (mainly pelargonidin, and cyanidin), and proanthocyanidins. Intakes of vitamin C (from food sources), total anthocyanidins, pelargonidin, and total flavonoids from food sources were each significantly associated with reduced incidence of Alzheimer’s dementia when controlled for age, sex, education, physical activity, participation in cognitive activities, and Apo-ɛ4 status (Table 2). However, cyanidins and proanthocyanidins intake were not associated with Alzheimer’s dementia risk (Table 2). Total vitamin C (food + supplement) was also not associated with Alzheimer’s dementia risk (HR for highest quartile compared to the lowest quartile was 0.78 (95% CI: 0.56 to 1.11; *p* for trend = 0.34). Participants in the highest quartile intake of pelargonidin (the primary anthocyanidin contained in strawberries) had a 44% reduced risk of Alzheimer’s dementia compared to those in the lowest quartile of intake (Figure 2). Further adjusting these models for cardiovascular conditions did not change the effect estimates for vitamin C (Q4 vs. Q1: HR = 0.63, 95% CI = 0.44 to 0.90, *p* trend = 0.01), total anthocyanidins (Q4 vs. Q1: HR = 0.69, 95% CI = 0.48 to 0.99, *p* trend = 0.25) pelargonidin (Q4 vs. Q1: HR = 0.63, 95% CI = 0.43 to 0.92, *p* trend = 0.01), and total flavonoids (Q4 vs. Q1: HR = 0.67, 95% CI = 0.45 to 0.98, p trend = 0.03) from food sources. Additionally, we investigated if the associations of vitamin C and flavonoids intake from foods were confounded by intake of other important antioxidant nutrients associated with cognition, i.e., vitamin E. The effect estimates for all the nutrients analyzed remained similar except that for total flavonoids (Table 2). However controlling for vitamin E and total flavonoids together in the model changed the effect estimates for vitamin C from foods and Alzheimer’s dementia association to marginal significance (Q4 vs. Q1: HR = 0.72, 95% CI = 0.49 to 1.0, *p* trend = 0.07). Similarly, the effect estimates of total flavonoids were modified when controlled for vitamin C and vitamin E together in a model (Q4 vs. Q1: HR = 0.69, 95% CI = 0.47 to 1.02, p trend = 0.05). However, the effect estimates for pelargonidin (Q4 vs. Q1: HR = 0.64, 95% CI = 0.44 to 0.94, *p* trend = 0.02) did not change when further adjusted for these nutrients.

We investigated the potential modification of the observed associations by age (>80 years/≤80 years), sex, education (>15 years/≤15 years) and APOE-ɛ4 status in basic-adjusted models. The only significant interaction effects were for total anthocyanidins and total flavonoids. The association of anthocyanidins with Alzheimer’s dementia risk occurred only among those who were APOE-ɛ4 negative (Q4 vs. Q1: HR = 0.46, 95% CI = 0.29 to 0.73) and not in those without the allele (Q4 vs. Q1: HR = 1.5, 95% CI = 0.79 to 3.0). The protective association of total flavonoid intake with Alzheimer’s dementia was present only in men (Q4 vs. Q1: HR = 0.22, 95% CI = 0.07 to 0.65) and not in women (Q4 vs. Q1: HR = 0.37, 95% CI = 0.53 to 1.26). 

## 4. Discussion

In this prospective study of older community adults, strawberry intake was associated with a 34% reduced risk of Alzheimer’s dementia compared to no or rare intake. Strawberries are rich in a number of flavonoid bioactives (e.g., anthocyanidins, mainly pelargonidin) that we found were each associated with lower Alzheimer’s dementia risk. Pelargonidin intake, which is a primary bioactive present in strawberries, appears to account for the protective effect of strawberries on Alzheimer’s dementia even when controlled for various confounding factors including demographics, genetic predisposition to dementia, lifestyle and other dietary factors including other antioxidant nutrients intake. 

To our knowledge, this is the first longitudinal cohort study of community older adults to report on the association of strawberries with Alzheimer’s dementia risk. Our results are supported by findings from the Nurses’ Health study in which strawberry consumption, anthocyanidins, and total flavonoid intake were each associated with slower cognitive decline [17]. Additionally, a randomized placebo-controlled trial of mixed berry beverage reported improved working memory [13]. A number of previous studies found that total flavonoid intake was associated with slower cognitive decline or reduced dementia risk [9,10,17,32]. There are mixed findings for vitamin C intake and risk of Alzheimer’s dementia, with some finding protective associations [10,33] and others no association [7,8]. We did not find any association between total vitamin C intake from food as well as supplements and Alzheimer’s dementia risk. Whereas, the association of vitamin C from food sources with Alzheimer’s dementia risk was found to be confounded by total flavonoids intake. 

The potential mechanistic links for the beneficial effect of strawberries may be due in part to the neuroprotective properties of the bioactive in strawberries. In animal models, strawberry consumption improved cognitive function, increased neurogenesis, and insulin-like growth factor-1 signaling, reversed neuronal aging by reducing oxidative stress and ameliorated metal-induced neurotoxicity [12,14,34]. Bioactives in strawberries, including pelargonidin, anthocyanidins, and total flavonoids, are known to have anti-inflammatory and antioxidant properties [35,36,37]. In vitro and in vivo evidence indicate that flavonoids, anthocyanidins, and some of their metabolites can cross the blood–brain barrier [38,39] and may directly act on neurons and glia via signal transduction cascades [40]. Anthocyanidin supplementation in an Alzheimer’s disease mouse model resulted in improved spatial working memory, attenuated oxidative stress post-intervention via PI3K/Akt/Nrf2 (phosphorylated-phosphatidylinositol 3-kinase/Akt/nuclear factor erythroid 2-related factor 2) pathways and restored pre-synaptic proteins, suggesting one role of anthocyanidin may be in protecting against neurodegeneration [41]. Pelargonidin is the most abundant anthocyanidin found in strawberries. Administration of pelargonidin in an Alzheimer’s disease rodent model demonstrated improvement in memory deficit and reduction in hippocampal oxidative stress [42]. It has been shown that various bioactives present in strawberries are absorbed, extensively metabolized, and circulated in the plasma of older adults [43]. Thus, the bioactive present in strawberries independently as well as synergistically may protect brain health in aging.

The current study has a number of important strengths including the longitudinal study design and 6.7 years of mean follow-up, the community-based sample of older adults, dietary assessment using a comprehensive and validated tool for older adults, structured clinical assessment and standardized diagnostic criteria for Alzheimer’s dementia, and assessment of many health and lifestyle factors associated with Alzheimer’s dementia. However, there are some limitations, as well. This is an observational study design so we cannot establish the temporal relationship and there can be some unaddressed confounding biases too. Our dietary assessment tool had one berry question just for the intake of strawberries. With no other berry question, there is a possibility of misreporting by those consuming other berries (blueberry, raspberries, or cranberries) also. Additionally, the study results may not be generalized to younger adults and nonwhite or Hispanic population.

## 5. Conclusions

Consumption of strawberries may reduce the risk of Alzheimer’s dementia in older adults, probably due to neuroprotective action of pelargonidin, anthocyanidins, and total flavonoids. Once replicated in other cohorts and confirmed by a clinical trial, these findings may have important public health implications, as the addition of strawberries to one’s diet can be an easy adaptation for older adults to reduce Alzheimer’s dementia risk.

## Figures and Tables

**Table 1:** Baseline characteristics by category of strawberry consumption among 925 Rush Memory and Aging Project participants, 2004–2018.

	Total (*n* = 925)	Strawberry Consumption		
		Rarely *n* = 243	1–3 Times/Month *n* = 428	>= Once/Week *n* = 254
Age, y, mean	81.16 ± 7.2	81.9 ± 7.1	81.2 ± 7.03	80.4 ± 7.4
Female, %	75%	71%	77%	75%
Education, y, mean	14.9 ± 2.9	14.6 ± 3.0	15.1 ± 3.1	15.0 ± 2.6
APOE-ɛ4 status, %	21.5%	23%	21%	21%
Cognitive activities, mean frequency	3.2 ± 0.6	3.1 ± 0.6	3.2 ± 0.7	3.3 ± 0.6
Physical activities, mean hours/week	3.4 ± 3.6	2.9 ± 3.6	3.3 ± 3.6	3.9 ± 3.8
Other Fruits Intake, servings/week	14.3 ± 7.3	12.9 ± 7.0	13.8 ± 7.0	16.4 ± 7.8
Total calories, kcal/day	1733 ± 539	1628 ± 555	1729 ± 518	1843 ± 538
Total flavonoid intake (mg/day)	223.2 ± 142.2	204.3 ± 150.0	218.0 ± 140.0	250.0 ± 135.0
Total vitamin E intake (mg/day)	71.3 ± 108.0	64.5 ± 99.0	76.6 ± 122.0	68.7 ± 89.4
Cardiovascular conditions, % present				
Hypertension	69%	69%	70%	68%
Myocardial Infarction	16%	18%	15%	15%
Diabetes	13%	12%	13.5%	13%
Stroke	11.5%	13%	11%	11%

**Table 2:** Association between strawberry consumption, vitamin C, and various flavonoid intake with Alzheimer’s dementia risk over the mean 6.5 years of follow-up among 925 participants of Rush Memory and Aging Project, 2004–2019.

Strawberry and Nutrients		Model 1: Basic (*n* = 925)	Model 2: Basic + Total vitamin E intake’ (*n* = 925)
**Strawberry Intake**			
	**Mean/Median Intake**	**HR (95% CI)**	
Continuous variable *	0.64 servings/week	0.76 (0.60, 0.96)	0.76 (0.61, 0.96)
Quartile Intake of Vitamin C and Flavonoids from Food Sources **			
**Vitamin C Intake**			
**1**	56.0 mg/day	Ref	Ref
**2**	98.4 mg/day	0.84 (0.59, 1.20)	0.85 (0.60, 1.22)
**3**	135.8 mg/day	0.77 (0.53, 1.10)	0.79 (0.55, 1.14)
**4**	199.5 mg/day	0.64 (0.45, 0.92)	0.67 (0.47, 0.97)
***p* for trend**		0.01	0.03
**Pelargonidin Intake**			
**1**	0.00 mg/day	Ref	Ref
**2**	0.89 mg/day	0.84 (0.60, 1.19)	0.83 (0.59, 1.17)
**3**	1.73 mg/day	0.78 (0.52, 1.04)	0.75 (0.53, 1.06)
**4**	3.90 mg/day	0.63 (0.43, 0.92)	0.65 (0.44, 0.94)
***p* for trend**		0.02	0.02
**Cyanidin Intake**			
**1**	0.30 mg/day	Ref	Ref
**2**	0.74 mg/day	0.72 (0.51, 1.02)	0.74 (0.52, 1.04)
**3**	1.22 mg/day	0.84 (0.60, 1.19)	0.87 (0.61 1.22)
**4**	2.20 mg/day	0.71 (0.49, 1.01)	0.74 (0.52, 1.07)
***p* for trend**		0.13	0.22
**Total Anthocyanidin Intake**			
**1**	4.41 mg/day	Ref	Ref
**2**	8.74 mg/day	0.60 (0.42, 0.85)	0.61 (0.43, 0.87)
**3**	14.0 mg/day	0.67 (0.48, 0.95)	0.68 (0.49, 0.97)
**4**	32.94 mg/day	0.69 (0.48, 0.99)	0.70 (0.49, 1.00)
***p* for trend**		0.23	0.26
**Total Proanthocyanidins Intake**			
**1**	29.83 mg/day	Ref	Ref
**2**	57.82 mg/day	0.72 (0.50, 1.04)	0.75 (0.52, 1.07)
**3**	84.28 mg/day	0.83 (0.59, 1.18)	0.86 (0.61, 1.22)
**4**	126.63 mg/day	0.73 (0.51, 1.06)	0.78 (0.56, 1.13)
***p* for trend**		0.19	0.32
**Total Flavonoid Intake**			
**1**	86.10 mg/ day	Ref	Ref
**2**	151.80 mg/day	0.94 (0.67, 1.32)	0.97 (0.69, 1.37)
**3**	227.05 mg/day	0.98 (0.69, 1.39)	0.99 (0.70, 1.41)
**4**	395.50 mg/day	0.67 (0.46, 0.98)	0.69 (0.47, 1.02)
***p* for trend**		0.04	0.05

* Basic model for strawberry intake is adjusted for age, sex, education, physical activity, participation in cognitive activities, Apo-ɛ status, dietary intake of other fruits, and total calorie intake. ** All the nutrients are calorie adjusted and the basic models for the nutrients are adjusted for age, sex, education, physical activity, participation in cognitive activities, and Apo-ɛ4 status. Model 2 was adjusted for basic model + calorie adjusted Vitamin E.

## Supplementary Materials

The following are available online at https://www.mdpi.com/2072-6643/11/12/3060/s1, Table S1: Baseline characteristics of Analytical sample (n = 925) and Overall cohort (n = 2152), Table S2: Correlation of baseline dietary intake of strawberry, vitamin C, other bioactive i.e., including Cyanidin, Pelargonidin, total Anthocyanidins, proanthocyanidins and total flavonoid from food sources.
