# Supplementary material for: Association of Strawberries and Anthocyanidin Intake with Alzheimer’s Dementia Risk
Source: Nutrients. 2019 Dec 14;11(12):3060. doi: 10.3390/nu11123060 (PMC6950087; doi:10.3390/nu11123060)
Supplement: Supplementary file 1 [file nutrients-11-03060-s001.pdf]

Supplementary Table S1: Baseline characteristics of Analytical sample (n=925) and Overall cohort (n=2152)

Variable	Analytical Sample (N=925)	Overall Cohort (n=2152)
Age, y, mean	81.16 ± 7.2	80.04 ± 7.6
Female, %	75 %	74 %
Education, y, mean	14.9 ± 2.9	14.8 ± 3.3
APO-E status, %	21.5 %	24.0 %
Cognitive activities, mean frequency	3.2 ± 0.6	3.2 ± 0.7
Physical activities, mean hours/week	3.4 ± 3.6	3.3 ± 3.6

Supplementary Table S2: Correlation of baseline dietary intake of strawberry, vitamin C, other bioactive i.e. including Cyanidin, Pelargonidin, total Anthocyanidins, proanthocyanidins and total flavonoid from food sources

	**Vitamin C**	**Cyanidin**	**Pelargonidin**	**Anthocyanidins**	**Proanthocyanidins**	**Total Flavonoids**
**Strawberry Intake**	0.25<br><.0001	0.42<br><.0001	0.94<br><.0001	0.37<br><.0001	0.39<br><.0001	0.27<br><.0001
**Vitamin C**	1.00	0.35<br><.0001	0.33<br><.0001	0.25<br><.0001	0.37<br><.0001	0.36<br><.0001
**Cyanidin**		1.00	0.49<br><.0001	0.39<br><.0001	0.83<br><.0001	0.45<br><.0001
**Pelargonidin**			1.00	0.42<br><.0001	0.47<br><.0001	0.32<br><.0001
**Anthocyanidins**				1.00	0.51<br><.0001	0.46<br><.0001
**Proanthocyanidins**					1.00	0.68<br><.0001
**Total Flavonoids**						1.00
